# Entanglements of Macromolecules and Their Influence on Rheological and Mechanical Properties of Polymers

**DOI:** 10.3390/molecules29143410

**Published:** 2024-07-20

**Authors:** Andrzej Pawlak, Justyna Krajenta

**Affiliations:** Centre of Molecular and Macromolecular Studies, Polish Academy of Sciences, 90-363 Lodz, Poland; justyna.krajenta@cbmm.lodz.pl

**Keywords:** entanglements, rheology of polymers, mechanical properties, semi-crystalline polymers

## Abstract

Flexible macromolecules easily become entangled with neighboring macromolecules. The resulting network determines many polymer properties, including rheological and mechanical properties. Therefore, a number of experimental and modeling studies were performed to describe the relationship between the degree of entanglement of macromolecules and polymer properties. The introduction presents general information about the entanglements of macromolecule chains, collected on the basis of studies of equilibrium entangled polymers. It is also shown how the density of entanglements can be reduced. The second chapter presents experiments and models leading to the description of the movement of a single macromolecule. The next part of the text discusses how the rheological properties change after partial disentangling of the polymer. The results on the influence of the degree of chain entanglement on mechanical properties are presented.

## 1. Introduction

Macromolecules based on carbon–carbon bonds in the main chain are long and flexible, allowing them to take on different configurations in space, from a straightened linear to an entangled coil. In the polymer, each macromolecule is surrounded by a number of other macromolecules. Because of this, macromolecules can interpenetrate and form topological entanglements, usually in the form of a loop of one macromolecule around another macromolecule [1]. The presence of entanglements, acting as physical knots in the macromolecular network, limits the possibilities for movement of macromolecules, as well as their relaxation. It is not surprising that the presence of entanglements affects many polymer properties, among them rheological and mechanical [2]. In particular, because they are present in the molten polymer, in the polymer solution, and in the amorphous phase of the solid polymer. The exception is the crystalline phase of the polymer, where the crystal structure does not allow entanglements to be accommodated and they are rejected outside the crystal volume.

The presence of entanglements was discovered many years ago in rheological studies. Berry and Fox [3] observed that the dependence of zero shear viscosity η_0_ on the molecular weight of the polymer *M* changes from a certain molecular weight. It is proportional to η_0_∼M, for low molecular weights and as η_0_∼M^3.4^ for higher molecular weights. The reason for this change is the entanglement of longer macromolecules.

The entanglements were also identified in solid amorphous materials, which above a glass transition can exhibit large deformation and elastic response. Such behavior is explained by the stretching and relaxation of the network of entangled macromolecules. Amorphous polymer or the amorphous component of semi-crystalline polymer at elevated temperatures behaves similarly to rubber. However, in rubbers, the macromolecular network is the result of chemical cross-linking. The entanglements act as physical cross-links, equivalent to chemical cross-links in rubber [4]. The influence of entanglements in the melt and in the elastic solid polymer on the properties can be described by equations of similar form. In the case of melt rheology, the relevant equation is of the form:

(1) $$G_{N}^{0}=\frac{g\rho RT}{M_{e}}$$

where G_N_^0^ is the shear modulus in the rubbery plateau region, R is the gas constant, T is the temperature, ρ is the density of the polymer, g is a coefficient equal to 1.0 [5] or 0.8 [6] and M_e_ is the molecular mass between the nodes of network, in this case between the entanglements.

A similar equation, based on the classical theory of elasticity [7], describes the high elastic properties of a solid polymer:

(2) $$G_{e}=\rho_{r}RT/M_{r}$$

where G_e_ is the shear modulus, $\rho_{r}$ is the density of the rubber, and M_r_ is the molecular mass between the network nodes [8,9,10]. There are other models that can be used to describe the properties, such as the phantom network model, leading to the modified Equation (2):

(3) $$G_{e}=\left(1-\frac{2}{f}\right)\frac{\rho_{r}RT}{M_{r}}$$

where f is the functionality, i.e., the number of polymer arms that are connected to a cross-linking junction [11].

From Equation (1), it can be concluded that the best parameter characterizing an entangled network of linear macromolecules is the molecular mass between the entanglements. The entanglement density, sometimes used instead of M_e_, is proportional to 1/M_e_. It is generally accepted that each type of polymer has its own M_e_ value. This value depends mainly on the architecture of the macromolecular chain. Calculations for monodisperse polybutadiene, polyisoprene, and polystyrene showed that M_e_ is independent of the weight average molecular weight M_w_ and is also independent of polydispersity (i.e., the M_w_/M_n_ ratio, where M_n_ is the number average molecular weight) [12]. These statements should be treated with caution due to the limited amount of data available. Some studies of mechanical properties show that in semi-crystalline polymers M_e_ can depend on M_w_ (see Section 4.1 for details). It was also found that M_e_ depends on the tacticity of the macromolecules [13,14]. Since entanglements may not be uniformly distributed along the macromolecule chain, the reported M_e_ values should be treated as averages.

There is a second parameter, important for characterizing entanglements, which is the critical molecular mass M_c_. When the molecular weight of the polymer exceeds this value, the relationship between the zero shear viscosity η_0_ and the molecular weight changes from η_0_~M_w_ to η_0_~M_w_^3.4^. M_c_ is greater than M_e_, and the ratio of their values is in the range of 1.0–3.5 [15].

Commercial polymers, typically processed, are in an equilibrium entangled state [13]. However, there are known methods to reduce entanglement. This allows for a deeper examination of the relationship between entanglement and polymer properties. Three groups of disentangling methods can be distinguished [16]: by dissolution and freezing (often called freeze-drying) [17,18,19,20,21,22], by polymerization with crystallization [23], by shearing of melt [24,25,26,27,28]. The dissolution is a method that can be used on a laboratory scale for any polymer. It is known that in a dilute polymer solution, the number of contacts between macromolecules, which then have the coil shape, decreases as the concentration of the solution decreases [29]. In a very dilute solution, one can even expect complete disentanglement and separation of the macromolecule chains. However, for practical reasons, it is usually better to have the polymer only partially disentangled, since chain entanglements are necessary to maintain the continuity of the amorphous phase of the solidified polymer under the action of force.

To use a polymer as a material, it is necessary to maintain the disentanglement state in the solid polymer. This can be performed by rapidly freezing the solution, for example using liquid nitrogen, and then removing the solvent, for example by sublimation [30,31,32,33,34]. There are variants of the solvent method in which disentanglement stabilization is achieved by crystallization from the hot solution or by adding a non-solvent to the frozen solution [35,36,37,38,39,40].

It is possible to disentangle the polymer during its polymerization [23,41,42,43]. Therefore, special catalysts and conditions are needed to separate the polymerization sites and enable immediate crystallization of the growing chains without entanglement. This approach has been mainly used for the polymerization of ultra-high molecular weight polyethylene (UHMWPE) [44,45,46,47,48,49].

Recently, a group of methods has been developed based on the observation that in polymer subjected to shear flow, there is not only orientation but also some disentanglement of macromolecules. In order to achieve disentanglement, the processing equipment has been modified to obtain different shear states, including pulsation or the addition of an elongation flow component [50,51,52,53,54,55]. This raises the prospect of commercial production of the partially disentangled polymers.

The next chapter briefly presents observations of the shape and dynamics of a macromolecule in the presence of other macromolecules, studied using neutron scattering, and a successful theoretical approach to describing the motion of macromolecule chains using a tube model.

Section 3 and Section 4 describe the rheological and mechanical properties of polymers from the perspective of entanglement research. Each section provides background based on the results of older experiments and results reported in the last few years. Most entanglement research has been focused on homopolymers. Not very often, especially when talking about entanglement reduction, polymer blends or polymer composites are analyzed.

## 2. Macromolecule in the Environment of Other Macromolecules

The plateau in the storage modulus G_N_^0^ observed for the molten polymer is similar to the plateau observed for cross-linked rubbers. This led to the conclusion that in the molten polymer, there is a network of macromolecules connected by nodes, which are entanglements. Therefore, research on the shape of macromolecules and the dynamics of chain motion in the molten and solid polymers was initiated. Almost simultaneously, the development of theoretical models, with the support of computer calculations, and the search for new experimental methods were addressed.

**Figure 1:**
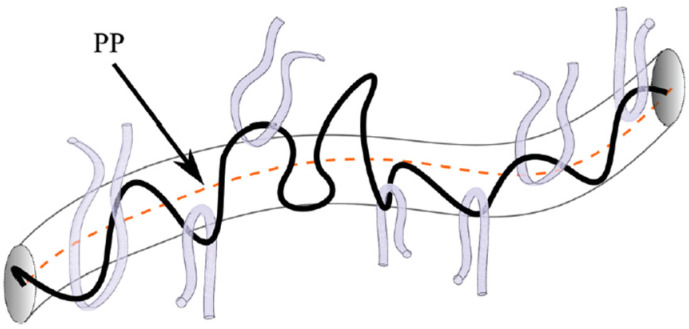
Virtual tube around the polymer chain. The centerline of the tube is referred to as primitive path (PP). The movement of chain in the direction of primitive path is possible, but transverse motion is restricted by the surrounding chains. Reproduced with permission from ref. [61]. Copyright 2020. Royal Society of Chemistry.

The results of rheological research have stimulated attempts to describe the observed behavior theoretically. One of the first widely known was the Rouse model of chain motion, based on the approximation of a chain by a set of balls connected by springs. This model successfully described the properties of low-molecular-weight polymers but was less effective for high-molecular-weight polymers [56]. The approach proposed by DeGennes was much more successful [57]. He proposed the description in which a macromolecule moves inside a virtual tube, created by other macromolecules, and the movement is similar to that of a snake (Figure 1). This is known as the reptation concept, and the descriptive model proposed by Doi and Edwards [58] is known as the tube model. The dynamics of the chain are characterized by the time of diffusion through the tube [59,60]. Using the model, it was possible to predict changes in η_0_ with M, but in the first version, an exponent of 3.0 was proposed instead of the experimentally found exponent of 3.4.

The development of the tube model by many authors has reduced this and other weaknesses of the model [6,59,62,63,64,65,66]. Modifications regarding contour length fluctuation [67], accounting for the dynamical variation of the primitive chain length [68], tube dilatation [69], and constraint release [70], accounting for the dynamics of the tube itself [68], have compensated for discrepancies between experimental results and the theory describing them. The success of the theory for the linear entangled polymers resulted in proposals to use the model for other architectures, such as mixtures of star and linear polymers [71], or long-chain branches [72]. The tube model is applied not only for polymer melts but also for the description of the dynamics of rubber elasticity [73]. The analysis of the reptation time τ_d_, i.e., time of diffusion along the tube length, shows that it is generally not short, since it depends on M^3.4^ through the zero shear viscosity η_0_ [74]:

τ_d_ = 20 M_e_ η_0_/(π^2^ R T ρ) (4)

When polyethylene has M = 4000 kg/mol, the reptation time is about 2 h [75], while reducing the molecular weight to M = 50 kg/mol shortens this time to 15 min. A more detailed description of tube model variants and their application can be found in the literature [76,77].

The search for experimental techniques suitable at the nanoscale focused on scattering methods. For dense polymers, light scattering, useful for studying dilute solutions, could not be used. Fortunately, small-angle neutron scattering (SANS) has just been developed to a level that made it possible to apply this method to the study of polymers. Neutron scattering results from the interactions of neutrons with the atomic nuclei. In the experiment, a collimated, monochromatic neutron beam hits the sample, and the scattering is monitored by a 2D detector [78]. The utility of SANS for polymer testing comes from both its coverage of large lengths and time scales and its ability to apply contrast to the polymer under study by introducing deuterated molecules [79].

The results of the first polymer studies using SANS were published in 1973. Early studies confirmed the random coil conformation of polymer chains in both molten and glassy polymers [80,81]. The SANS technique was used to determine the actual radius of gyration R_g_ of the chain in the bulk state. The dimension of macromolecules in a bulk polymer sample can be characterized by the value of the K coefficient, which depends on the molecular weight by K = (R_g_^2^/M_w_)^0.5^ [82]. Typical K values for polymers range from 0.028 to 0.046 nm·mol^0.5^/g^0.5^ [83]. This means, for example, that molten polyethylene with M_w_ = 10^5^ g/mol has R_g_ = 14 nm. Calculations show that a single macromolecular coil has a density of less than 1% of the volume density of the polymer, which indicates that the empty space inside it is filled with fragments of other macromolecules [84].

Quasi-elastic neutron scattering can be used to study the dynamics of polymers at different length scales [85]. At the large-scale dynamics, the observable processes are the chain diffusion, the reptation, and the Rouse dynamics. These processes control the rheological properties of the polymer [86]. The corresponding length scale where the topological confinement caused by interpenetrating coils dominates is above 10 nm. Typical time scales for processes involving the entire macromolecule are nanoseconds or more [79]. Studies of polyethylene using an ultra-high-resolution spectrometer have confirmed that the observed changes in the dynamic structure factor are consistent with the reptation model [87]. The subject of studies with the application of contrast is not only the dynamics of individual chains in the melt but also their fragments, such as ends or branch points [88].

Nowadays, there is a tendency to increase the complexity of the investigated system [86,89]. Computer simulations, such as molecular dynamic simulation, are very helpful in the interpretation of scattering results [90]. The polymer dynamics in nanocomposites in the presence of movement confinement is an example of modern research with the use of the neutron spin echo technique [78,91,92,93]. Schneider et al. [94], studying poly(ethylene propylene) with silica, found that the confinement length, i.e., the tube diameter, decreased with silica contents, but the entanglement density was reduced at high nanofiller content. Nusser et al. [95] analyzed two relaxation processes associated with reptation, i.e., constraint release and contour length fluctuations.

From the above brief review of the literature, it appears that the SANS data support the tube model of entangled macromolecule motion.

## 3. Rheological Properties of Molten Polymers

### 3.1. Methods for Characterizing Entanglements

**Figure 2:**
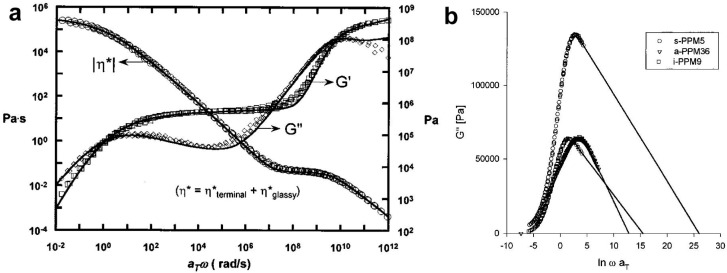
(**a**) Master curves for polylactide (PLA) sample showing nearly 14 decades in frequency obtained via time–temperature superposition. η* is the complex viscosity, which is the sum of terminal (η*_terminal_) and glassy (η*_glassy_) components. Reproduced with permission from ref. [98]. Copyright 2005. The Society of Rheology. (**b**) Example of using the integral method to calculate the G_N_^0^ modulus from the area under the G″ peak, when some data are unavailable. The curves represent three polypropylenes. ω × a_T_ is a reduced angular frequency. Reproduced with permission from ref. [13]. Copyright 1998. American Chemical Society.

To describe the entanglement state using Equation (1), it is necessary to know the rheological moduli, obtained by measuring linear viscoelastic properties in an oscillatory shear experiment, performed over a wide range of frequencies and temperatures. In this experiment, the storage modulus G′ and the loss modulus G″ are usually determined. The G_N_^0^ modulus in Equation (1), necessary to calculate M_e_, is the value of the storage modulus at the frequency at which the loss modulus reaches its minimum [12]. This is shown in Figure 2a. Unfortunately, this classic approach often cannot be applied due to a lack of data for some high frequencies or simply the absence of a visible G″ minimum. For this reason, a number of other methods have been developed to characterize entanglements [12,13,96,97]. For example, if the minimum of the G″ modulus is poorly visible, as in the case of polydisperse polymers, the minimum of the tan δ = G″/G′ value can be taken as the position in the frequency to read G_N_^0^, but this criterion is more arbitrary [12,97].

An approach for determining M_e_ that is increasingly used is to calculate G_N_^0^ using the numerical integration of the area over the terminal relaxation peak of G″(ω). This method, sometimes called the integral method, can be used for monodisperse polymers. The equation useful in G_N_^0^ calculation has the form [99]:

(5) $$G_{N}^{\;0}=2\pi \int_{-\infty }^{\infty }G^{\prime \prime }(\omega )dln\omega$$

In many cases, however, data about G″ are missing at high frequencies. For this reason, it was proposed to use a linear approximation for the missing data and then perform calculations in accordance with Equation (5) [13]. The application of the numerical integration to various polypropylenes is shown in Figure 2b.

A similar approach has been proposed for highly polydisperse materials, in which the terminal relaxation spectrum is broad, and the loss modulus peak is usually not recorded in its entirety. Therefore, assuming the symmetric shape of the G″ peak, calculations can be performed by taking a doubled peak area from the origin to the frequency corresponding to the maximum [12,97,100].

The method, now called the maximum method, was by Raju et al. [101]. If the chain dispersion is narrow and chains are long, the plateau modulus G_N_^0^ can be calculated from equation:

G_N_^0^ = 3.56 G″_max_ (6)

where G″_max_ is the maximum of G″ modulus.

**Figure 3:**
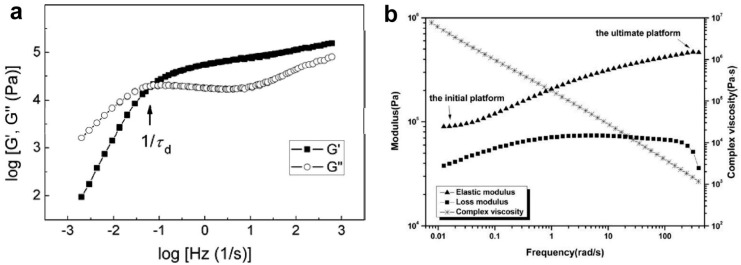
(**a**) Storage and loss modulus as a function of frequency at 150 °C of the bulk polystyrene (PS) sample. τ_d_ is the linear viscoelastic relaxation time. Reproduced with permission from ref. [18]. Copyright 2012. American Chemical Society. (**b**) Results of a dynamic frequency sweep experiment of a disentangled ultra-high molecular weight polyethylene (UHMWPE) sample, performed at temperature of 160 °C. There is no cross-over point. Reproduced with permission from ref. [104]. Copyright 2015. Springer Nature (Berlin, Germany).

For many polymers, the presence of a cross-over point where G′ and G″ are equal is observed [18] (Figure 3). Some researchers prefer to characterize entanglements by specifying this cross-over point, especially in the case of high polydispersity or when the polymer is semicrystalline [12]. It should be mentioned that the inverse values of the dynamic moduli at their intersection G′ = G″ characterize the polydispersity of the samples [102] and that the cross-over frequency shifts to lower values with molecular weight [103]. To determine the level of entanglements, attempts are being made to use both the cross-over modulus G_x_ and the cross-over frequency ω_x_.

Wu [97] and Nobile and Cochini [105] stated that the relationship between the cross-over modulus G_x_ and the plateau modulus G_N_^0^ should have the general form of:

log (G_N_^0^/G_x_) = f(M_w_, M_n_, M_z_) (7)

where M_w_, M_n_, M_z_ are weight-averaged, number-averaged, and z-averaged molecular weights, respectively. When the polydispersity of polymer is lower than 3, the Equation (7) has the form [97]:

log (G_N_^0^/G_x_) = 0.38 + 2.63 log(M_w_/M_n_)/(1 + 2.45 log(M_w_/M_n_)) (8)

According to Equation (8), the cross-over modulus is a measure of entanglement. Higher values of G_x_ mean a higher density of entanglements.

**Figure 4:**
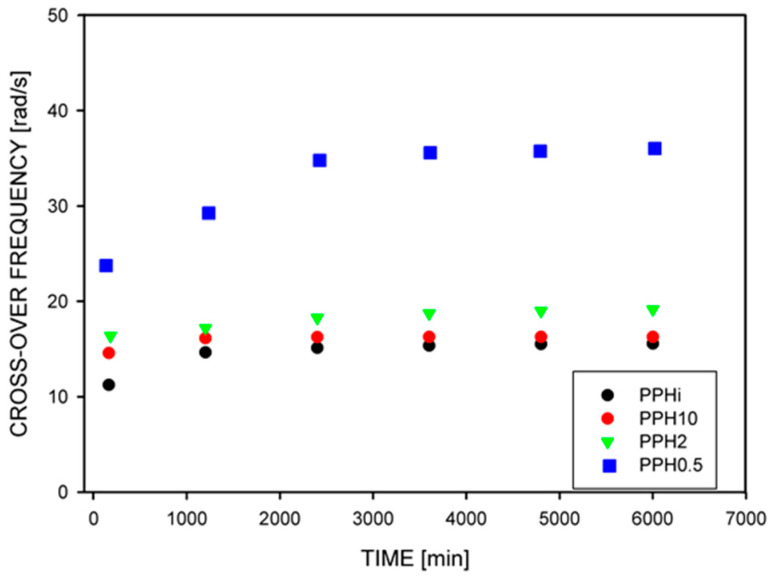
Changes in the cross-over frequency with annealing time at 185 °C. PPHi means equilibrium entangled polypropylene. PPH10, PPH2, and PPH0.5 are partially disentangled polypropylenes obtained by dissolving in xylene with concentration of 10, 2, and 0.5 wt.%, respectively. Reproduced with permission from ref. [106]. Copyright 2019. Elsevier (Amsterdam, The Netherlands).

Krajenta et al. [106] analyzed changes in the cross-over frequency as a function of the annealing time for differently entangled polypropylenes (PP) (Figure 4). It was observed that at the beginning of annealing, a higher frequency corresponded to a lower density of entanglements. This was consistent with the equation proposed by Martins et al. [107] linking ω_x_ with the number of entanglements per macromolecule Z = M/M_e_:

1/ω_x_ ≈ τ_R_ Z (9)

where τ_R_ is the Rouse relaxation time, which depends on the chain length to the second power [108]. 1/ω_x_ is equal to the reptation time [12,109]. As the annealing time increased, the cross-over point shifted toward higher frequencies, reaching a plateau. This would be interpreted as re-entanglement if, surprisingly, this was not also observed for equilibrium-entangled PP. The reason for the behavior observed with annealing time is not clear, because from other experiments for the same materials, the time of re-entanglement for the most disentangled PP was about 2 h, not 45 min, after which the plateau visible in Figure 3 was reached [106].

It seems that the cross-over modulus, rather than the cross-over frequency, can be used to compare changes in the entangled state of a single polymer when other methods are not available. Sometimes the cross-over point does not exist (see Figure 3b), as in the case of a certain type of disentangled UHMWPE studied by Li et al. [104]. The absence of the cross-over point was interpreted as the absence of entanglement.

The molecular masses between entanglements, determined for equilibrium entangled polymers by the methods described above, are summarized in Table 1. For comparison, information on the critical molecular masses is also included. Two conclusions about M_e_ can be drawn from the data in Table 1. First, there is a scattering of results obtained in different laboratories. Second, the attempt to find a universal relationship between tacticity and M_e_ has not been successful. It is also worth noting that many polymers have been characterized so far.

Methods for determining M_e_ using Equation (1) or Equation (5) are also applied to characterize partially disentangled polymers in the melt state. The correctness of such application may be a matter of debate, but so far there are no arguments against it.

### 3.2. Entanglements in Polymer Blends

Table 1 shows data characterizing the entanglements of homopolymers. However, entanglements also occur in blends of two or more polymers. The entanglement in the blend can be determined using rheological methods. In immiscible blends, it is assumed that the degree of entanglement is the same as in the polymers used. In the miscible blend, entanglement depends on the composition and the entanglement density of each blend component [126,127,128]. It has been observed that chemically dissimilar chains entangle more than similar chains, so their contribution to the total number of entanglements is large [126,129]. The contribution of different types of entanglements to the average entanglement density in a blend ν_e_, or to the average molecular mass between entanglement in a blend M_e_ has the form:

(10) $$\nu_{e}=\frac{\rho }{M_{e}}=\frac{{\varphi_{1}}^{2}\rho_{1}}{M_{e1}}+\frac{{\varphi_{2}}^{2}\rho_{2}}{M_{e2}}+\frac{2\varphi_{1}\varphi_{2}\rho_{1}\rho_{2}}{M_{e12}}$$

where ρ is the density of the blend, ρ_1_ and ρ_2_ are the densities of the blend components, φ_1_ and φ_2_ are the volume fractions of the components, M_e1_ and M_e2_ are the molecular masses between the entanglements of the same polymer, and M_e12_ is the molecular mass between the entanglements of macromolecules of two polymers [129].

**Figure 5:**
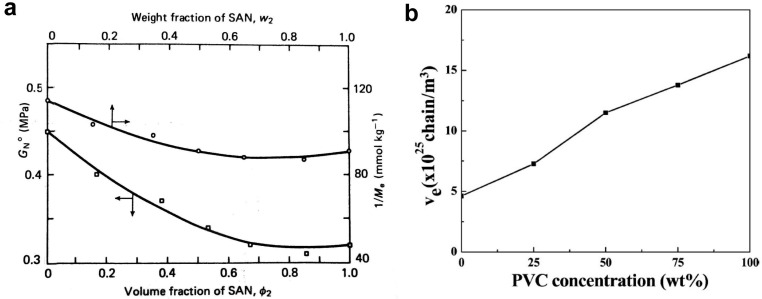
(**a**) Plateau modulus G_N_^0^ as a function of the volume fraction φ_2_ of SAN in the PMMA blend, and 1/M_e_ versus weight fraction w_2_ of SAN. The arrows on the curves show their corresponding axes. Reproduced with permission from ref. [130]. Copyright 1987. Elsevier. (**b**) Entanglement density, ν_e_, as a function of PVC content in PVC/α-MSAN blends. Reproduced with permission from ref. [126]. Copyright 2013. American Chemical Society.

The rheology of compatible blends of poly(methyl methacrylate)(PMMA) and poly(styrene-acrylonitrile)(SAN) was investigated by Wu [130]. The G_N_^0^ modulus for the blend with 60–90% SAN was lower than the moduli of both components, which means that M_e12_ > M_e1_ and M_e12_ > M_e2_ (Figure 5a). An explanation has been proposed that the specific interchain interaction of dissimilar polymers tends to locally align the chain segments, stiffening the chains, and reducing the convolution, resulting in reduced entanglement between dissimilar chains. Other blends studied by Wu [131] were PMMA/poly (ethylene oxide), PMMA/poly(vinylidene fluoride), and polystyrene/poly(phenylene oxide). For these blends, the G_N_^0^ modulus was always between the values for the pure blend components, although a negative deviation from the straight line was visible.

An example of the multicomponent blends studied was that described by Song et al. [126]. They analyzed miscible blends of poly(vinyl chloride) (PVC)/poly(α-methylstyrene-acrylonitrile) (α-MSAN) toughened with polybutadiene-g-poly(styrene-co-acrylonitrile)(PB-g-SAN). The entanglement density as a function of PVC content was calculated according to Equation (1), based on the results of rheological measurements of G′. An approximately linear relationship was found (Figure 5b).

Song et al. [132] also examined miscible blends of polyphenylene oxide/polystyrene (PPO/PS) toughened by high-impact polystyrene (HIPS) and polybutadiene-graft-polystyrene (PB-g-PS) copolymer. The authors assumed that the blends had an equilibrium density of entanglements, proportional to the composition. It was observed that mechanical properties, i.e., impact strength and elongation at break, gradually increased with the assumed entanglement density of the matrix.

The properties of UHMWPE were modified by blending with PP [133]. A significant reduction in the apparent melt viscosity was observed, and for the blend with 20% of PP, the entanglement density, ν_e_, determined from the storage modulus on the rubbery plateau, was 181 mol/m^3^, i.e., lower than that of UHMWPE, which was 433 mol/m^3^. Adding 1 wt.% poly(ethylene glycol) to the UHMWPE/PP blend further reduced ν_e_ to 147 mol/m^3^.

### 3.3. Partially Disentangled Melts

**Figure 6:**
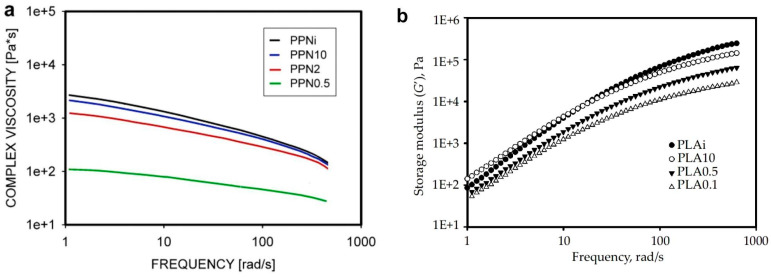
(**a**) Complex viscosity of polypropylene as a function of frequency and disentangling. PPNi –fully entangled polypropylene with M_e_ = 9900 g/mol, PPNi10—polypropylene with M_e_ = 12,600 g/mol, PPN2—polypropylene with M_e_ = 18,000 g/mol, PPN0.5—polypropylene with M_e_ = 19,100 g/mol. Reproduced with permission from ref. [106]. Copyright 2019. Elsevier; (**b**) Storage modulus G′ as a function of frequency, measured at 180 °C. PLAi –fully entangled polymer with M_e_ = 10,500 g/mol, PLA10—partially disentangled PLA with M_e_ = 16,300 g/mol, PLA0.5—PLA with M_e_ = 32,800 g/mol, PLA0.1—PLA with M_e_ = 65,300 g/mol. Reproduced with permission from ref. [117]. Copyright 2020 by Lukasiewicz Research Network—Industrial Chemistry Institute.

The development of methods for disentangling macromolecules has opened the possibility of deeper exploration of the role of entanglements. Studies usually involve the comparison of entangled and partially disentangled polymers. The frequency sweep test is often performed using a rotational rheometer to demonstrate the change in G′ and G″ moduli and the change in complex viscosity. Figure 6a illustrates the change in complex viscosity as a function of frequency, measured for the same polypropylene, but disentangled to different extents [106]. PPNi is a fully entangled polymer with M_e_ = 9900 g/mol, and PPN0.5 is the most disentangled polymer with M_e_ = 19,100 g/mol. The decrease in viscosity values with disentanglement is visible, as is the reduction in G′ at high frequencies (Figure 6b) in measurements of differently entangled polylactide (PLA) [117].

A decrease in the plateau modulus was found when metallocene-catalyzed high-density polyethylenes (HDPEs) were compared with commercially available HDPE [114]. This was due to a reduction of entanglements during metallocene-catalyzed polymerization. The effect of polymerization conditions on the entanglement of ultra-high molecular weight polyethylene (UHMWPE) was studied using rheological amplitude sweep and time sweep tests by Pandey et al. [23] and Chen et al. [134]. The conclusion was that slower polymerization led to fewer entanglements per unit chain length. When UHMWPE polymerization was carried out in the presence of polyhedral silsesquioxane (POSS), more entanglements in the polymer were obtained because POSS limited crystallization and created conditions for entanglement of growing chains instead of their participation in crystallization [135].

Drakopoulos et al. [136] studied the relaxation dynamics of UHMWPE using torsional rheology and observed different γ-relaxation behavior of disentangled and entangled polymer, suggesting that the entanglement density affects the free volume and thus affects the γ-relaxation. The analysis of the dynamic viscosity of cereal starches was conducted by Guo et al. [137,138]. The samples were prepared from aqueous solutions with concentrations above or below the critical concentration, which was determined based on measurements of the change in the slope of specific viscosity as a function of concentration. This critical concentration varied significantly (3.3–16.9%, g/l), depending on the type of starch. The cross-over frequency was used to characterize the starch rheology, except for the samples prepared from 1 wt.% solutions, for which no cross-over point was observed, which was explained by the very low density of interchain entanglement in these samples.

The subject of studies in the last decade has also been the rheology of nanocomposites [139,140,141,142,143]. Several authors observed that when nanoparticles were well dispersed in the polymer matrix, the melt viscosity of the system was reduced, unlike composites with micro-sized fillers or nanofiller agglomerates, in which the viscosity increased [142,144]. The effect of viscosity reduction depends on the size of the nanoparticle, which should be smaller than the mesh size of the matrix [140]. Sufficiently small particles can increase the free volume of the melt, thereby reducing chain entanglement and accelerating the relaxation of the molten matrix. Examples of nanocomposites whose rheology was studied were poly(methyl methacrylate)-silicon [142], polystyrene–polyoxymetalate [140], UHMWPE-POSS [144], UHMWPE-TiO_2_ [145].

### 3.4. Re-Entangling of Polymers

**Figure 7:**
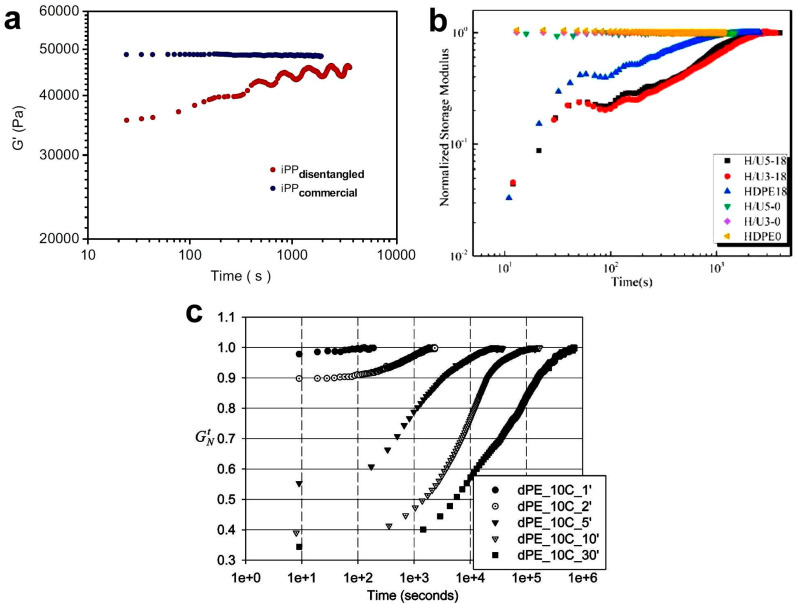
(**a**) Changes in the G′ modulus as a function of time, measured at 180 °C on entangled and disentangled PP. Reproduced with permission from ref. [37]. Copyright 2009. Elsevier. (**b**) Buildup of normalized storage modulus for HDPE/UHMWPE blends at 200 °C. The H/U5-18 designation means HDPE with 5% added UHMWPE sheared at rate of 18 s^−1^. Reproduced with permission from ref. [149]. Copyright 2020. Elsevier. (**c**) Results of dynamic time sweep test performed at 160 °C for five UHMWPEs synthesized at low temperature with different polymerization times (1′–30′), leading to different entanglements. G′_N_ is the elastic modulus normalized by maximum plateau modulus in the modulus buildup. Reproduced with permission from ref. [23] Copyright 2011. American Chemical Society.

Many of the disentangled polymers were studied using a rheological time sweep test. The goal was to show how moduli and viscosity change over time [43,106,135,146,147,148,149,150,151]. This experimental interest resulted from the expectation that after a sufficiently long time at high temperature, the macromolecules would re-entangle, i.e., the polymer would be in equilibrium again and the rheological parameters would reach their initial values (Figure 7a). Research has shown that this does not often occur within a reasonable experimental time. Many authors treated achieving a constant value of the G′ modulus as a criterion for reaching the equilibrium entangled state, even if the value of this modulus was lower than initially. The results were then presented in normalized form, i.e., as the ratio of the actual G′ value to the constant G′ value at the end of the test (Figure 7b).

Regardless of the definition used, the time after which the disentangled polymer reached constant values of rheological parameters was long and depended on the molecular weight of the polymer. For example, for linear polyethylene, it increased from 100 s to 11 h when the molecular weight increased from 9.0 × 10^4^ to 3.6 × 10^6^ g/mol [152]. Even longer times, e.g., 55–118 h, were measured for UHMWPE with M_w_ = 5 × 10^6^ g/mol [23,153] (Figure 7c). The proposed explanation for the long times observed is that as the molecular weight increases, the number of entanglements per chain also increases, so it takes longer to reach a thermodynamically stable state. Talebi [60] found that the relationship between the time of re-entangling of UHMWPE, t, and its molecular weight M_w_ is:

ln t = a × 2.8(ln M_w_) (11)

where a is a constant.

The influence of polymerization conditions, and therefore disentanglement, on rheological properties were observed by Rastogi et al. [154], Chen et al. [134], and Ronca et al. [43]. The reduction of entanglement time resulting from melt annealing of UHMWPE was found by Li et al. [104]. The reason was the increased chain mobility at high temperatures. The dependence of the entanglement time on the density of entanglements was not confirmed in the study of two UHMWPEs by Chammingkwan et al. [48]. However, careful analysis showed that the most likely cause was the presence of voids in the entangled polymer, which reduced contact of the polymer with the rheometer plates.

Sometimes, during time sweep experiments, it is observed that the increase in modulus is not smooth, but undulations, i.e., periodic fluctuations, are visible (see Figure 7a) [37,147,149]. The reasons for this phenomenon have not yet been explained and it cannot be ruled out that there are technical, instrumental causes of measurement disturbances.

Based on the shape of the G′ curves (Figure 7c), Pandey et al. [23] suggested that the re-entanglement process consists of two stages, with a rapid increase in G′ modulus and entanglements in the first phase and much slower entanglement in the second phase. For a semicrystalline polymer, Li et al. [135] associated the first phase with the process of explosive disintegration of crystals, analogous to the observations of the melting of single-crystal polyethylene mats carried out by Barham and Sadler [75]. The second phase of re-entanglement should be controlled by the reptation movement of the macromolecule chains. Similar explanations were also proposed by Pandey et al. [23] and Talebi [60]. A serious objection to this interpretation is the fact that the time of the first phase in the case of polyethylene is, according to the publication by Li et al. [135], as long as 6 h (see the dPE_10C_5′ curve in Figure 7c), which is much longer than usual crystal melting time. Rather, it appears that the melting of the crystals introduces a less entangled phase of the melt into the interior of the also partially disentangled melt derived from the amorphous phase. The equalization of the entanglement density, faster in the less entangled phase and slower in the more entangled phase, may be the cause of the macroscopically observed changes in moduli. Two stages of re-entanglement were also observed by Fu et al. [147]. In their opinion, these two stages differ in the type of entanglements that arise. In the initial phase (approximately 100 s for HDPE), simple entanglements (twists, knots, links) are formed, while in the second phase, loops are formed.

The time sweep test was used to investigate poly(L-lactide) re-entanglement. The polymer was disentangled by freeze-extraction from solutions of various concentrations [19]. The G′ modulus was lower for polylactide obtained from a less concentrated solution, which was consistent with expectations regarding the level of disentanglement. The time to reach equilibrium value G′ was longer for the less entangled polymer.

**Figure 8:**
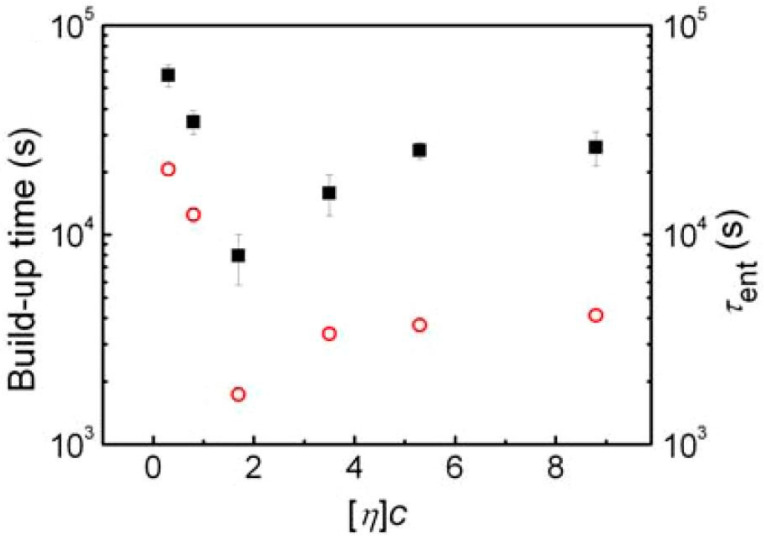
Equilibrium entanglement time of PS samples freeze-dried from solutions with different initial concentrations. The filled black squares refer to the modulus buildup time at which G′(t)/G_N_^0^ = 0.98, and the open red circles represent the calculated times of the entanglement recovery. Reproduced with permission from ref. [18]. Copyright 2012. American Chemical Society.

Another polymer for which entanglement restoration was studied was polystyrene [18]. Depending on the solution from which the polystyrene was obtained, starting from low concentrations, the re-entanglement time first decreased, while for higher concentrations the trend was opposite, i.e., the re-entanglement time increased (Figure 8). The behavior observed below a concentration of 3.0 in Figure 8 was as expected because more separated chains needed more time to entangle. For the high-concentration region, the explanation has been proposed that a more concentrated solution results in a smaller chain coil size and more compact globules after freeze-drying. Therefore, it takes more time for the coils to envelop the neighbors.

The re-entanglement has also been examined for PP [106]. It was found that after 2 h of melt annealing, the viscosity of PP still remained below the initial level, although during this time the mechanical properties typical for entangled PP were restored. It was observed that the re-entanglement time depended on the initial degree of entanglement, i.e., it was longer for the less entangled polymer, as well as on the molecular weight of PP. At larger M_w_, re-entanglement required a longer time.

**Figure 9:**
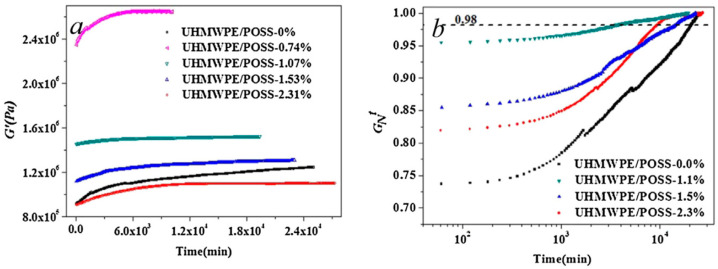
(**a**) Results of a dynamic time sweep test performed at 170 °C for UHMWPE with different POSS content. (**b**) Data from Figure 9a shown after normalization. G_N_^f^ is the storage modulus normalized by the maximum recorded value. Reproduced with permission from ref. [135]. Copyright 2014. Elsevier.

The rare case is the study of composites in which the polymer has reduced entanglement. An example is UHMWPE containing POSS nanoparticles introduced in various concentrations during polymerization [135]. The change in G′ over time is shown in Figure 9a, and the same data after normalization in Figure 9b. The highest initial storage modulus was found for a POSS content of 0.74%, and it decreased at higher POSS content. This was explained as a result of the highest molecular weight of this UHMWPE, which was polymerized with low filler content. Similarly to UHMWPE, the re-entanglement process in the UHMWPE/POSS nanocomposite required an extremely long time, exceeding 1000 min.

The partially disentangled PP composites with graphene nanoplatelets (0.1–4 wt.%) were prepared by the melt shear, and changes in the rheological properties of the melt were observed with time [155]. It was measured that partially disentangled neat PP tested at 200 °C needed 2000 s to reach the viscosity of fully entangled PP. Much shorter times of only 100 s were measured for the composites, however their final viscosities were at a lower level than for the entangled PP. The authors presented the opinion that the disentangled state remained at the observed level because the nanoplatelets, due to their strong interaction, limited the movement of the chains. Doubts regarding the given explanation are raised by the fact that a similar effect was observed for some homopolymers.

Luo et al. [156] studied the re-entanglement of chains in a PP micro-composite with a high content (35 wt.%) of boron nitride. The disentanglement of the composite was achieved by steady-state shear. As expected, before shear was applied, the composite had a higher viscosity than PP. After shearing, the viscosity of the composite decreased below the PP level, but increased rapidly within the first 30 s after shear cessation and then remained stable at a level below the PP viscosity.

The build-up of entanglement as a function of time can also be characterized by measuring the normal force in the time sweep test performed with the rotational rheometer [19,60]. In the case of completely entangled UHMWPE, no changes in normal force were recorded, while in the case of disentangled polymer, a gradual decrease in normal force was observed over time. This was caused by a reduction in the free volume of the melt during the formation of entanglements.

The time sweep rheological test is most often used to characterize changes in entanglements with polymer residence time in the melt, but they can also be investigated using other rheological tests. For example, a strain sweep test and a frequency sweep test with multiple repeated cycles were used to characterize UHMWPE [104].

## 4. Mechanical Properties Dependent on the Entanglement of Macromolecules

### 4.1. Properties of Equilibrium Entangled Polymers

The presence of entanglements in an amorphous polymer or in the amorphous phase of a semi-crystalline polymer determines the mechanical properties of the entire polymer [157]. In both amorphous and semicrystalline polymers, the main deformation features, mainly examined in the tensile test, are the same. Under the action of force, the deformation is initially elastic, followed by yielding when segmental rearrangements are possible. With a larger deformation, strain softening may occur, resulting from localized plastic deformation leading to necking. The strain softening is usually not visible when results are presented as a true stress–true strain curve. As the strain increases, plastic flow is observed at an approximately constant stress level, followed by an increase in stress, which is known as strain hardening [158]. Based on the behavior of deformed polystyrene and polycarbonate, it can be shown that the strain hardening is more pronounced when the polymer is more entangled [159]. At large deformations, orientation and stretching of macromolecules with their partial disentanglement are observed [160].

**Figure 10:**
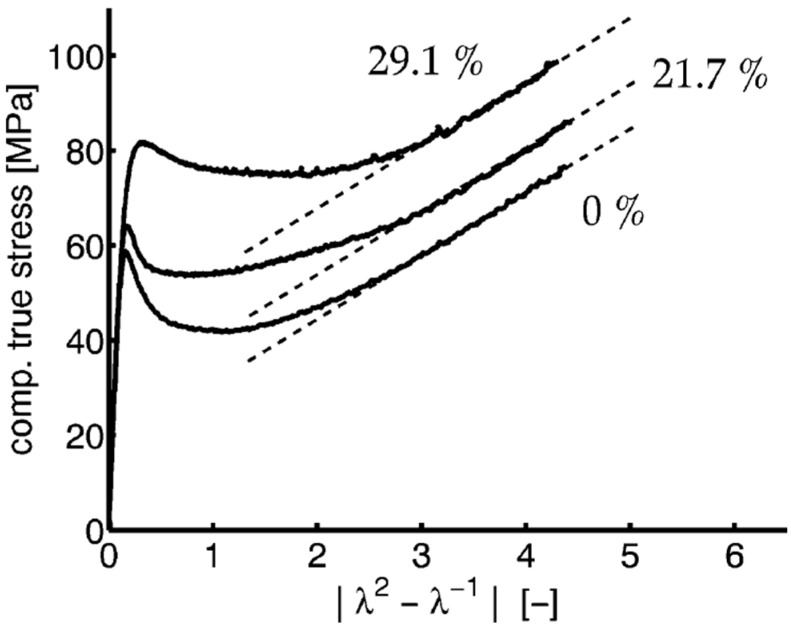
Compressive behavior of isothermally crystallized poly(ethylene terephthalate) samples with different degrees of crystallinity. λ is the extension ratio. Reproduced with permission from ref. [158]. Copyright 2004. American Chemical Society.

The deformation phases described above are observed when the polymer is ductile, and only some of them when the polymer is brittle. The same polymer can become brittle or ductile depending on the temperature and the strain rate. The irreversible plastic deformation of amorphous polymers occurs through the formation of shear bands or crazes. Essentially the same deformation phases are visible when the polymer is compressed [158] (Figure 10).

**Figure 11:**
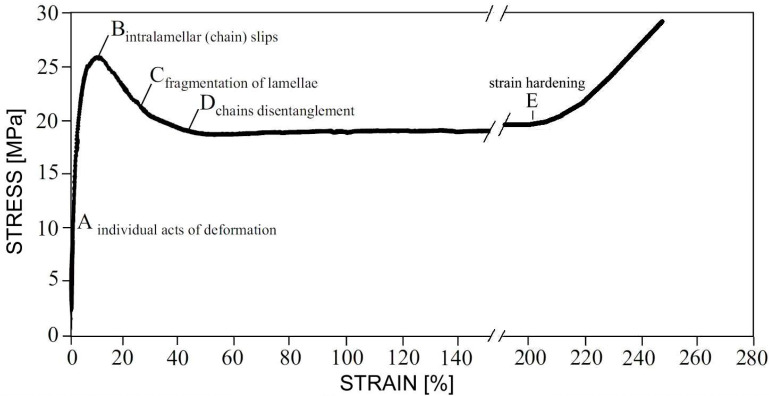
The stress–strain relationship for high-density polyethylene deformed in tension. The characteristic deformation points are marked A–E. Previously published experimental data [164,165] were used to prepare the figure.

The deformation of a semicrystalline polymer analyzed at the micro-scale is more complicated because its structure contains crystalline lamellae connected by macromolecules [4]. The two main types of connections are tie macromolecules, that is, macromolecules having fragments in neighboring crystals, and macromolecular entanglements [160]. Therefore, in semicrystalline polymers, the physical cross-links of the macromolecular network are both the entanglements in the amorphous phase and the crystals themselves [4]. The main stages of the evolution of the semicrystalline polymer structure in relation to the strain–stress dependence were described by Strobl [4,161,162,163]. At small strains (point A in Figure 11) single acts of deformation occur in the form of lamellar separation, rotation of lamellae, and interlamellar slips. At the yield point (B), massive chain slips are initiated. The amorphous chains gradually orient with increasing deformation. During the strain softening (C), lamellar fragmentation occurs, and fibrillation begins, i.e., the formation of fibrils by fragmented, oriented crystallites. Once a plateau is reached (point D), the chains begin to disentangle, allowing further deformation, with the neck propagation through the sample. At larger strains, the strain-hardening begins due to the stretching of the macromolecular network (E).

Men et al. [166] studied the deformation at point C. They changed the density of entanglements of selected polymers by blending them with other miscible polymers and analyzed the free shrinkage of these materials. It was found that stretching a semicrystalline polymer always results in the deformation of the interpenetrating networks of interlocked crystallites and the entangled amorphous phase. The deformation from point B is dominated by the stretching of the amorphous phase, controlled by entanglements.

Although entanglements are involved in all phases of deformation, attention is focused on the strain-hardening phase, when changes in the entropy of the entangled network occur, resulting from its stretching, orientation, and possible partial disentangling.

The plastic phase of deformation during polymer stretching can be described by an equation of the form [167]:

σ = σ_y_ + λ(NkTn^0.5^)/3 [λ(3 − λ^2^/n)/(1 − λ^2^/n) − 1/λ^2^(3 − 1/λn)(1 − 1/λn)] (12)

where σ is the true stress, σ_y_ is the yield stress, N is the number of entanglements per unit volume, n is the number of flexible units between entanglements, and λ is the extension ratio. When n is large or λ is small, the Equation (11) can be simplified and takes the form [168,169]:

σ(λ) = σ_y_ + G_R_(λ^2^ − 1/λ) (13)

where G_R_ is the strain-hardening modulus. G_R_ depends on the temperature T and the entanglement density ν_e_ [128,170]:

G_R_~ν_e_/T (14)

According to this equation, the slope of the strain-hardening phase, as proportional to the physical cross-links, should increase with the entanglement density [158]. Equations (12)–(14) do not contain M, therefore it is assumed that the strain-hardening and the entanglement density should be independent of the molecular weight. However, in the case of semi-crystalline polymers, due to the presence of a crystalline phase, tensile and compression properties suggest that there is an influence of molecular weight [158]. The reasons for this are discussed later in the text.

There is one more parameter characterizing the polymer network that depends on entanglements. This is the maximum draw ratio, related to the square root of M_e_ [171,172]. The entanglement network affects not only the shape of the stress–strain relationship but also the micro-mechanisms of deformation, such as crazing and/or shear yielding [173,174,175]. This issue is especially important for amorphous polymers.

**Figure 12:**
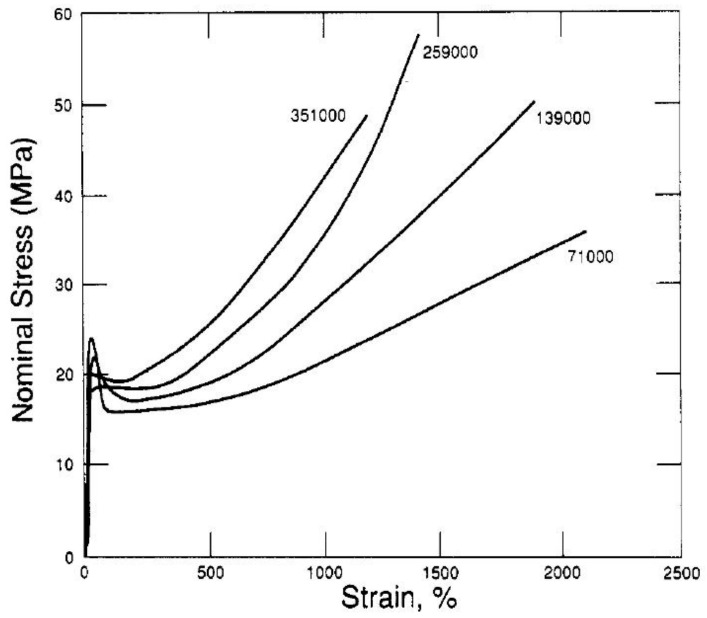
Nominal stress versus nominal strain for a series of rapidly quenched linear polyethylenes with the most probable molecular weight distributions (see numbers on curves). The strain hardening phase is clearly visible, earlier for higher molecular weight. Reproduced with permission from ref. [176]. Copyright 1994. American Chemical Society.

The relationships presented above (Equations (12)–(14)) result from both theoretical and experimental research [158,176,177]. The experiments confirmed that the strain hardening occurs earlier and is stronger when the density of entanglements is higher. Figure 12 shows the strain–stress relationship for a series of polyethylenes with different molecular weights [176]. The authors explained changes in the strain-hardening slope with a change in M_w_ as the result of an increase in the entanglement density with an increase in molecular weight. For the same reasons, a decreased deformation ratio was observed.

Schrauwen et al. [158] examined the properties of polyethylene, polypropylene, and poly(ethylene terephthalate) during uniaxial compression. One of their conclusions was that the strain-hardening depended on the sample preparation, i.e., slowly crystallized samples exhibited lower strain-hardening. According to the authors, this was due to the lower density of chains in the amorphous phase, resulting from the pulling out of the macromolecular chains during slow crystallization. The similarity of results for different polymers supported the opinion that the crystalline phase does not contribute to strain-hardening, which is controlled by the chain entanglement density. Schrauwen et al. [158] agreed with Kenendy’s [176] opinion on the influence of M_w_ on the chain entanglement density, explaining that the more difficult pulling out of chains during the crystallization of polymer with higher M_w_.

Polyethylenes with M_w_ in the range of 5 × 10^4^ to 5 × 10^6^ g/mol were examined by Bartczak and Kozanecki [177]. The experimental stress–strain curves were fitted using the Arruda and Boyce model [178]. The authors obtained a decrease in M_e_ from 1020 to 414 g/mol as M_w_ increased. The reason for the change in M_e_ was differences in the structure of the amorphous phase, resulting from a different course of crystallization. According to the authors, during the plastic deformation, partial disentangling of polymer chains occurs.

### 4.2. Properties of Disentangled Polymers

In the above-described experiments, the differences in entanglements resulted from the crystallization conditions, but in the 1970s it was already possible to study polymers disentangled in solutions. The influence of entanglement reduction on mechanical properties was found when Lemstra and Smith [179] performed experiments involving the spinning of polymer solutions after gelation. They observed that polyethylene gel could be spun to very high ratios (about 30 times) and create strong fibers with an elastic modulus of 108 GPa [179,180]. The reason for the excellent drawability of polyethylene was the reduction of entanglements in the gel [29].

**Figure 13:**
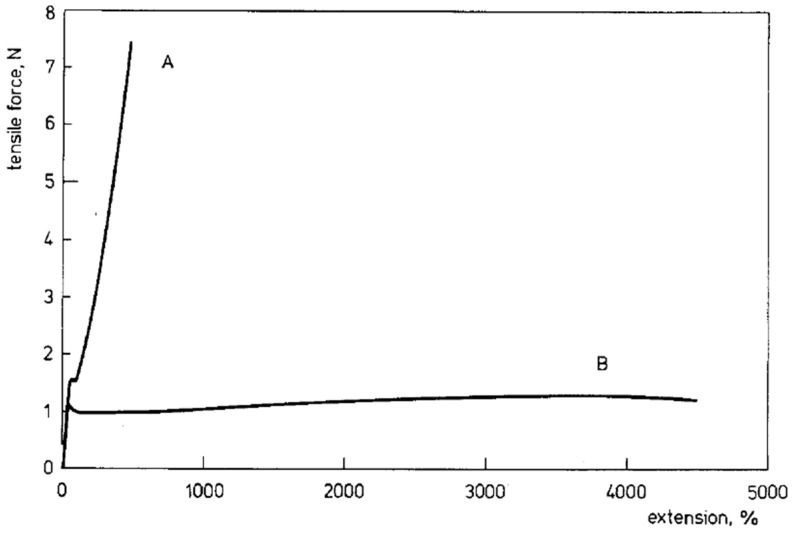
Load–extension curves measured at 120 °C for UHMWPE. A—melt crystallized film, B—film cast from 2 wt.% solution in decalin, with reduced entanglements. Reproduced with permission from ref. [179]. Copyright 1980. Springer Nature.

Smith and Lemstra also made a film by drying a UHMWPE gel and found that very high strains could be achieved, while the same polymer processed by melting showed much earlier breaking [179] (see Figure 13). The ultradrawing behavior of gel films was also studied by Yeh et al. [181]. A high deformation ratio of 60 was obtained for a film made of ultra-high molecular weight polypropylene (UHMWPP) prepared from a gel solution [38]. Similarly prepared UHMWPP sheets were subject to a two-step drawing by Ikeda et al. [182]. The samples for the second stage were cut from the sheets pre-drawn in the first stage. The tensile drawing of samples to break was performed at high temperatures of 130 °C or 150 °C. Based on the determined draw ratio of the neck and the maximum draw ratio of the sample, the authors concluded that both depend on the inverse root of the concentration of the solution from which the sheet was cast.

One of the earlier works using the freeze-drying disentangling method was the one published by Huang et al. [183], in which poly(ethylene terephthalate) obtained from the solutions with a concentration of 2–40 wt.% was drawn by solid-state co-extrusion at the temperature of 70 °C. The maximum extrusion draw ratio achieved depended on the concentration of the solution from which the polymer was obtained. At very low concentrations the density of entanglement was too low to ensure the continuity of the material, while at too high concentrations the entangled network prevented high drawability.

**Figure 14:**
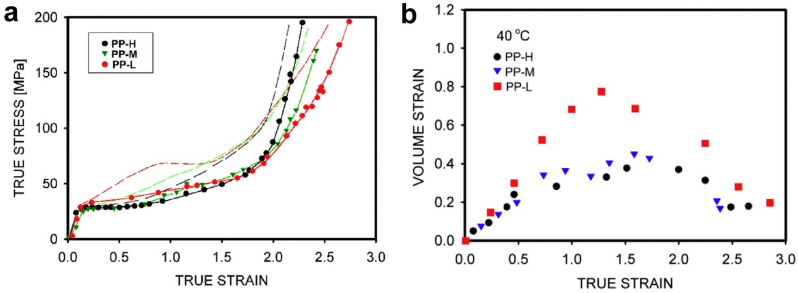
(**a**) True stress–true strain curves for polypropylenes deformed at 40 °C. PP-H is entangled polymer with M_e_ = 9.9 kg/mol, and PP-M and PP-L are partially disentangled polypropylenes with M_e_ = 14.5 kg/mol and M_e_ = 18.0 kg/mol, respectively. The strain-hardening, dependent on entanglement, is visible from the true strain of 1.9. The dashed lines show the shapes of curves after correction of the cross-section area and the strain for the presence of voids fraction in bulk samples; (**b**) The evolution of volume strain during deformation. The volume strain is a measure of the increase in sample volume due to the cavitation. Reproduced with permission from ref. [184]. Copyright 2018. Elsevier.

Studies of the tensile deformation of entangled and partially disentangled polypropylenes obtained by crystallization from solutions at temperatures 20–100 °C were carried out by Pawlak et al. [184]. The measured masses between entanglements were 9.9, 14.5, and 18.0 kg/mol. Mechanical properties before and at the yield did not change due to the reduction of entanglements, but the strain-hardening increased with the density of the molecular network formed by entanglements (Figure 14a). It is known that semi-crystalline polypropylene cavitates during tensile deformation [185]. However, for the first time in the case of polymers, it was observed that the reduction of entanglements intensifies cavitation (Figure 14b). The cavitation in disentangled polypropylene was possible not only at low temperatures, as is usually the case, but even when the tensile test was performed at the temperature of 100 °C.

The process of void generation during uniaxial stretching was modeled using the molecular dynamics method by Logunov and Orekhov [186]. They modeled the properties of amorphous polyethylene and concluded that the molecular entanglements slow down the growth of voids and their aggregation in the bulk of polyethylene.

The tensile properties of polycarbonate disentangled to varying degrees by using modified shear procedures during extrusion (by rotation, vibration, or together), were examined by Wang et al. [55]. Although the decrease in viscosity was confirmed rheologically, the most disentangled sample had tensile properties similar to those made from raw pellets. The reason for this was not discussed. The authors presented some conclusions regarding processability using the injection molding method. The lower processing temperature and pressure when the polymer was disentangled were beneficial for molding the products, obtaining lower residual stresses and therefore better (slightly) mechanical properties. The disentangled sample, having a reduced melt density, can be expected to show superiority over the original material when it becomes difficult to fill the mold during injection molding.

**Figure 15:**
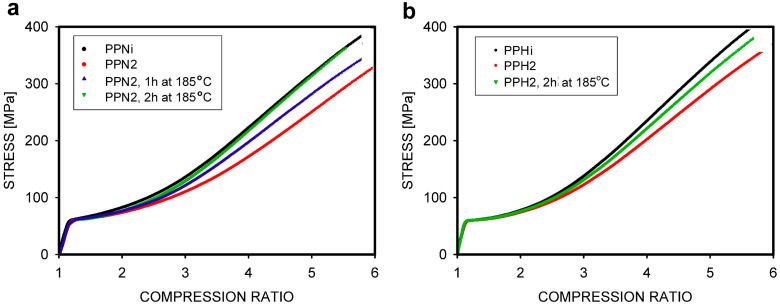
Mechanical properties of compressed polypropylenes with different entanglement densities: (**a**) PPNi—initial polypropylene with M_w_ = 250 kg/mol, PPN2 –the same polymer after disentanglement, non-annealed and annealed 1 h or 2 h; (**b**) PPHi—initial polypropylene with M_w_ = 400 kg/mol, PPH2 –the same polymer after disentanglement, non-annealed and annealed 2 h. Reproduced with permission from ref. [106]. Copyright 2019. Elsevier.

If the mechanical behavior in the strain-hardening phase depends on M_e_, a change in slope should be visible when the initially disentangled polymer is re-entangled by melt annealing. The solution-disentangled PP samples were annealed at different times and examined in a compression test by Krajenta et al. [106]. The choice of compression rather than extension was intended to avoid the potential impact of cavitation. Figure 15 shows that the stress–strain curves of the entangled sample and the annealed sample overlap after 2 h when the PP has M_w_ = 250 kg/mol, but 2 h of annealing was not sufficient for fully re-entangle the polymer with higher M_w_ = 400 kg/mol.

Although tensile testing is most commonly used to characterize mechanical properties, studies on impact behavior have also been performed. The Izod impact test was used to measure the strength of two sintered UHMWPEs, fully entangled and partially disentangled [187]. This less entangled polyethylene showed an impact strength of 80 kJ/m^2^, while the entangled PE showed an impact strength of only 70 kJ/m^2^. The unexpected result was probably due to better sintering of the grains in the sample with more mobile macromolecules.

In the UHWWPE research, Zhang et al. [188] used the Charpy version of the impact test. The commercial polymers used (SLL 5 and GUR 4150) had the same M_w_, so the molecular weight should not affect the impact strength. Tensile testing, a prelude to the main tests, showed stronger strain-hardening for SLL 5, which indicated more entanglements. This was also confirmed by rheology. In the impact measurements, the less entangled polyethylene had three times higher Charpy impact strength than the entangled polyethylene (144 kJ/m^2^ vs. 43 kJ/m^2^). The GUR 4110 sample showed a ductile fracture, while the SLL 5 samples fractured brittle. The authors interpreted the observed differences as related to the different structures of polymers, i.e., smaller nascent particles and a lower degree of entanglement, which led to a better connection of the polymer grains during film formation.

Other impact properties were tested on micro-sized spherical polystyrene projectiles [125]. The projectiles had different M_w_ in the range of 9.3–270.0 kg/mol, so one of them had a value below M_e_ = 13.4 kg/mol. The spheres were launched against a rigid substrate, which led to deformation at an ultra-high strain rate. During impact, the bottom of the projectile heated up and deformed in plastic flow. In the upper part, a localized shear banding with brittle fracture was observed for spheres from disentangled PS and extensive shear banding followed by crazing in the entangled projectiles. The absence of entanglements resulted in a rapid transition to fracture.

**Figure 16:**
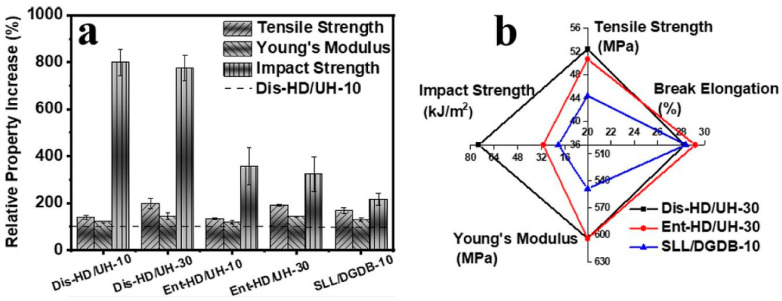
Comparison of the mechanical properties of synthesized HDPE/UHMWPE blends containing disentangled or entangled UHMWPE: (**a**) Improvement of the mechanical properties of blends in relation to the properties of HDPE, assumed as 100%. Dis-HD/UH-30 is the blend containing 30 wt.% disentangled UHMWPE, and Ent-HD/UH-30 is the blend containing 30 wt.% of entangled UHMWPE. Dis-HD/UH-10 is the blend containing 10 wt.% disentangled UHMWPE, and Ent-HD/UH-10 is the blend containing 10 wt.% of entangled UHMWPE. SLL/DGDB-10 is the commercial component blend containing 10 wt.% UHMWPE, shown for comparison. (**b**) Graphical presentation of the results for selected blends. Reproduced with permission from ref. [191]. Copyright 2023. American Chemical Society.

One way to improve the processability of UHMWPE, which is unsatisfactory due to M_w_ > 10^6^ g/mol, is to blend it with lower molecular weight polyethylene, which is the main component of the formed blend. Compatible high-density polyethylene (HDPE) or linear low-density polyethylene (LLDPE) macromolecules penetrate UHMWPE during blending, reducing its entanglements [189,190]. Chen et al. [191] prepared blends of HDPE with disentangled or entangled UHMWPE obtained directly in the reactor. With the same composition, the tensile properties, such as elastic modulus, elongation to break, and tensile strength, of the blends with the entangled and disentangled components were similar (Figure 16). The big difference was in the impact strength, where the partially disentangled samples showed 2–3 times more energy absorbed at the break. The reason was a change in the internal structure. Disentangling the chains promoted more efficient growth of oriented shish-kebab structures, which were also longer in partially disentangled material.

Similar observations regarding the promotion by disentanglement of the growth of more oriented shish structures, with more densely attached kebab-like lamellae, were made by Tao et al. [192]. In their three-component polyethylene blend, weakly entangled UHMWPE macromolecules acted as tie molecules and significantly improved the impact resistance.

**Figure 17:**
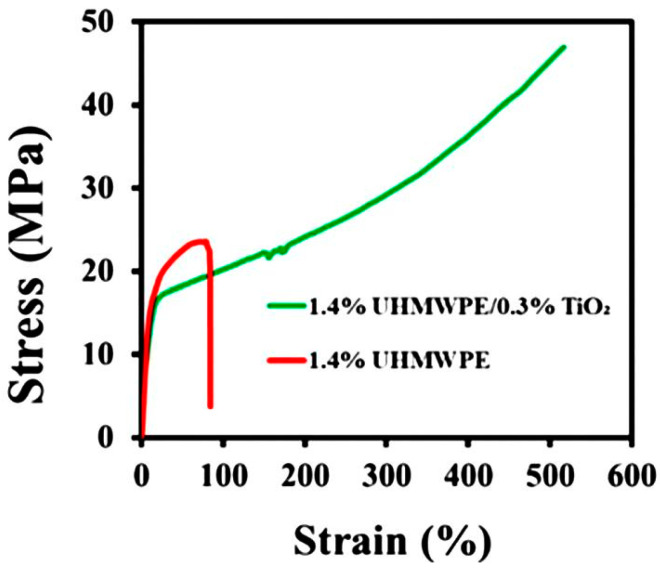
Tensile properties of UHMPE partially disentangled in a 1.4% wt. solution in decahydronaphtalate and a similarly prepared UHMWPE/TiO_2_ composite. Reproduced with permission from ref. [145]. Copyright 2023. Royal Society of Chemistry.

Although most studies of mechanical properties on reduced entanglements of macromolecules have been conducted using homopolymers, some studies have also been performed on polymer composites. The processability and tensile mechanical properties of UHMWPE were improved by adding 0.1–0.5 wt.% TiO_2_ nanoparticles into the dilute UHMWPE solution [145]. The dispersion of nanoparticles changed with their concentration, from homogeneous through clusters to aggregates. The viscosity of composites with dispersed TiO_2_ was lower than that of pure partially disentangled UHMWPE, reaching a minimum for content of 0.3 wt.% which meant a 50% decrease in entanglement. The presence of nanofiller increased the crystallinity by 7%, which together with the reduction in entanglement, influenced the mechanical properties. Figure 17 shows the drastic difference in the tensile properties of the partially disentangled UHMWPE and its composite.

The properties of disentangled PLA and its composites containing 0.1–1 wt.% of multi-walled carbon nanotubes were investigated in comparison to entangled materials in the tensile test [193]. The test samples were prepared by extrusion. Better dispersion of the filler was obtained inside the less entangled polypropylene. During the deformation of the less entangled homopolymer, the initiation of plastic deformation occurred at a lower yield stress, and the stress increased more slowly during the strain-hardening. In the composite, the strain-hardening was stronger for the less entangled polylactide due to better dispersion of the filler.

Smith and Lemstra’s [179] approach to forming fibers from a dilute solution, described above, was successful because the entanglement density in the polymer used was reduced. On this basis, Galeski developed the concept of producing all-polymer composites using the disentangled polymer to prepare fibers while mixing with a second polymer [194]. The mixing takes place in the extruder at a temperature higher than the melting point of the polymer for matrix, but lower than the melting temperature of the disentangled polymer. When shear stresses are applied during mixing, the grains of the disentangled polymer are oriented and form fibers. At the end of mixing, when the temperature decreases, an all-polymer composite is obtained.

**Figure 18:**
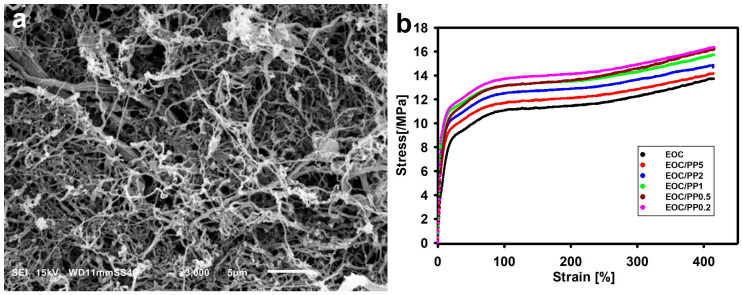
(**a**) Morphology of polypropylene fibers exposed after dissolving the EOC/PP composite in heptane. The fibers were formed from partially disentangled polypropylene, obtained from 0.2 wt.% solutions in xylene. (**b**) Tensile properties of EOC and composites with 4 wt.% of PP. The numbers in the description indicate the concentration of solution from which the partially disentangled PP was obtained. A lower number corresponds to a lower density of entanglements. Reproduced with permission from ref. [195]. Copyright 2024. John Wiley and Sons (Hoboken, NJ, USA).

An example of the application of this concept was the preparation of a composite of ethylene–octene copolymer (EOC) and polypropylene with a composition of 96:4 wt.% [195]. Previously disentangled PP powders were used to prepare composites by extrusion. Microscopic observation confirmed that a network of PP fibers was formed inside the EOC during extrusion (Figure 18a). This resulted in increased stresses during tensile deformation compared to pure EOC (Figure 18b). The increase in stress was greater in the case of PP obtained from a more dilute solution, from which thinner fibers were formed, giving at the same content a larger contact area between components, and thus better reinforcing the composite matrix.

## 5. Concluding Remarks

Until a few years ago, the disentangling of macromolecules was performed almost exclusively in the laboratory, and the goal was to better understand the impact of entanglements on the properties of polymers. Now that the entanglement-limited polymerization process has been mastered and it is possible to disentangle polymers during extrusion, we can think about the production of commercial polymers with limited entanglements, most likely useful in special applications.

From the point of view of processing and subsequent applications of the polymer, rheological and mechanical properties are particularly important. Rheological studies show that the disentangling of macromolecules leads to a decrease in the viscosity of polymer melt and a decrease in modulus values. The behavior of polymers at the micro level has already been quite well described by the tube model. One conclusion is that the time for the macromolecule to disentangle itself from the tube is quite long. This can be seen when performing experiments on the re-entanglement of macromolecules. However, polymer re-entanglement requires additional research, because existing descriptions of the phenomena raise doubts. It should be explained why there are two phases of the process and why the final modulus values are in many cases lower than expected. There is a lack of a more complete description of the rheological behavior of polymer blends and composites containing partially disentangled polymer. It has been reported that during composite preparation, melt mixing of the disentangled polymer has a beneficial effect on the dispersion of the nanofiller.

The mechanical properties are as important as the rheological properties. There is a consensus that during the tensile test, usually performed, but also during the compression test, the presence of entangled macromolecules has the greatest impact on the strain-hardening phase of deformation. The stress buildup and strength of the tested polymer depend on the entanglement network. Few research results indicated a positive change in impact properties due to partial disentanglement of macromolecules. This seems to be due to the improved consistency of the tested material, obtained during the processing of a polymer characterized by increased macromolecular mobility. Research on mechanical properties has so far focused on homopolymers. Experiments are necessary to show how the properties of composites change, for example as a result of better dispersion of the reinforcement.

## Figures and Tables

**Table 1:** Molecular masses between entanglements and critical molecular masses.

Polymer	M_e_ [g/mol]	M_c_ [g/mol]	Literature Sources
Polyethylene	830–2600	2800–3480	[13,15,97,110,111,112,113,114]
Poly(ethylene oxide)	1600–2200	5870	[15,97,112]
Polyisobutylene	6900–10,500	13,100–17,000	[15,97]
Polylactide	4000–10,500	9000–10,000	[98,115,116,117]
Syndiotactic polylactide	11,800		[118]
Isotactic polylactide	4100		[118]
Ultra-high molecular weight polypropylene	6300		[99]
Atactic polypropylene	4390–7050		[13,15,112,119,120]
Syndiotactic polypropylene	1700–3400		[13,112]
Isotactic polypropylene	5500–9900		[13,99,106,111,112]
Atactic poly(methyl methacrylate)	10,000–13,600	29,500	[15,112]
Syndiotactic poly(methyl methacrylate)	5800–9200		[97,112]
Isotactic poly(methyl methacrylate)	14,600		[97]
Syndiotactic polystyrene	14,500		[121]
Isotactic polystyrene	17,500–28,880		[15,97,112,121]
Polycarbonate	1330–1660		[112,119]
Poly(ethylene terephtalate)	1170–1450		[15,97,119]
Poly(methylene oxide)	2110–2640		[15,97,119]
Poly(ethylene-2,5-furanoate)	3500		[122]
1,4- polybutadiene	1850		[123]
Poly(phenylene oxide)	3150–3620		[97,119]
Poly(tetrafluoroethylene)	5580		[97]
Poly(vinylidene fluoride)	2400		[97]
Poly(methyl acrylate)	9070		[97]
Poly(vinyl acetate)	9100–11,400	24,500	[15,97]
Poly(caprolactam)	2490		[97]
Poly(dimethylsiloxane)	8160–12,000	24,500	[15,97]
Atactic poly(1-hexene)	12,100		[124]
Syndiotactic poly(1-hexene)	14,100		[124]
Isotactic poly(1-hexene)	15,200		[124]
Atactic polystyrene	13,400–18,700	38,000	[15,97,112,113,121,125]

## Data Availability

No new data were created.
